# Antibacterial Potential of Extracts and Phytoconstituents Isolated from *Syncarpia hillii* Leaves In Vitro

**DOI:** 10.3390/plants11030283

**Published:** 2022-01-21

**Authors:** Muthukuttige M. N. Perera, Satish N. Dighe, Peter L. Katavic, Trudi A. Collet

**Affiliations:** Innovative Medicines Group, Faculty of Health, Queensland University of Technology, 60 Musk Avenue, Kelvin Grove, QLD 4059, Australia; muthukuttige.perera@hdr.qut.edu.au (M.M.N.P.); pakatavic@gmail.com (P.L.K.)

**Keywords:** extract, phytochemicals, *Syncarpia hillii*, antimicrobial

## Abstract

(1) Background: Rapidly increasing antibiotic resistance is one of the greatest threats to global health, affecting individuals regardless of age. Medicinal plants are widely used in traditional medicine to prevent and attenuate infectious conditions with minimal adverse effects. However, only a few have been phytochemically investigated for their medicinal properties and subsequent biological activities. *Syncarpia hillii,* a plant traditionally used by Indigenous Australians to treat sores, wounds, and skin infections, is no exception. (2) Methods: Primary extracts obtained from mature *S. hillii* leaves were evaluated for their antibacterial potential against 19 bacterial strains. The methanol extract was subjected to compound isolation and identification due to its preliminary bactericidal efficacy. (3) Results: *Staphylococcal* species were the most susceptible bacterial strain with a MIC value of 0.63 mg/mL to the *S. hillii* methanol extract. Quercetin-3-O-β-D-glucuronide and shikimic acid isolated from *S. hillii* methanol leaf extracts exhibited enhanced antibacterial effects against the tested bacteria with quercetin-3-O-β-D-glucuronide eliciting a MIC value of 0.78 µg/mL against *E. faecalis*. (4) Conclusions: *S. hillii* leaves are comprised of bioactive compounds that are bactericidal against several Gram-positive and Gram-negative bacteria.

## 1. Introduction

Plants are a well-known source of active metabolites and, hence, form the basis of numerous pharmaceutical products [1,2]. At present, 80% of the world’s population uses medicinal plants to treat basic illnesses, mostly as extracts or isolated active compounds [3].

Indigenous Australians are known for using a diverse range of plant species for the treatment of different ailments; however, only a few have been investigated for their medicinal properties [4]. *Syncarpia hillii*, a plant used in Australian traditional medicine [5] that grows primarily in southeast Queensland and, more predominantly, Fraser Island [6,7], has been shown to contain very different essential oils from other species of the same genus [8]. Using steam distillation and other analytical techniques, the oil was found to be comprised of 53–80% hillyl acetate, 6–12% hillone [9], 22% α-pinene, a smaller quantity of monoterpenes such as α-thujene, *p*-cymene, and terpinen-4-ol, and several sesquiterpenes [9]. Nevertheless, apart from traditional knowledge of Indigenous Australian communities regarding the use of *S. hillii* for treating wounds and skin-related infections [5], only a minimal number of studies pertaining to the pharmacological effects of the plant have been undertaken [7,9,10].

Multi-drug-resistant microorganisms and their associated biofilms have become a global challenge [11], as the majority of antibiotics currently in use were discovered prior to 1970 [12]. Therefore, the discovery and development of new antimicrobial drugs with novel modes of action are essential to control the emergence of multi-drug-resistant pathogens [13].

As such, the aim of this current study was to evaluate the antimicrobial potential of primary extracts obtained from *S. hillii* leaves against 19 Gram-positive and Gram-negative bacteria and subsequently isolate and identify the bioactive phytochemicals responsible for the bactericidal effect. The methanol extract was further tested for biofilm eradication activity due to its superior antibacterial potential compared to other primary extracts against methicillin-resistant *Staphylococcus aureus* (MRSA). Upon phytochemical analysis, two compounds were isolated from the *S. hillii* primary methanol extract and also evaluated for their antimicrobial activity. Overall, this is the first study to report the detailed extraction, isolation, and bactericidal efficacy of leaf-derived extracts and compounds from *S. hillii*.

## 2. Results

### 2.1. Antibacterial Effects of Primary Methanolic Extracts

Aqueous, methanol, ethanol, isopropanol, and hexane extracts obtained from *S. hillii* mature leaves at a concentration of 100 mg/mL were tested against 19 bacterial strains using the WDA (Table 1). Comparatively, the ethanol and methanol extracts demonstrated the highest level of inhibition and were shown to be bactericidal against all Gram-positive and four Gram-negative bacteria. However, the inhibitory effect of both extracts did not exceed the ZOI elicited by the standard antibiotic control for any of the bacteria tested. The inhibitory effect of both the methanol and ethanol extracts were almost identical, although the methanol fraction was marginally superior with regard to antimicrobial activity against Gram-negative bacteria, i.e., *P. vulgaris*, *P. mirabilis*, and *A. baumannii*. Moreover, the ZOI produced by aqueous (6.56 ± 0.83) and methanol (6.33 ± 0.45, *p* < 0.0001) extracts against the clinical isolate of MRSA (ATCC 33591) was only slightly less compared to the antibiotic control (7.00 ± 0.00). In general, a higher antimicrobial activity was demonstrated in those fractions extracted with polar solvents compared to nonpolar solvents.

### 2.2. Minimum Inhibitory Concentration (MIC) of Primary Extract

MIC values were determined for all five *S. hillii*-derived extracts against the bacterial strains, which were shown to be susceptible from the previous WDAs (Table 2). MIC values for the tested extracts ranged from 0.63 to 10 mg/mL. Methanolic and ethanolic extracts produced the lowest MIC values compared to the other extracts, although none were shown to impede the growth of *E. faecalis*, *E. faecium*, or *E. gallinarum* at a concentration of 10 mg/mL. Further, none of the bacteria screened were susceptible to hexane extracts at the highest concentration tested.

### 2.3. Minimum Bactericidal Concentration (MBC) of Primary Extracts

From the MIC studies, methanolic and ethanolic extracts were identified as the most potent. Hence, both were selected to determine MBC values, which ranged from 5 to 20 mg/mL (Table 3). Of all the bacteria tested, *Staphylococcal* strains were found to be most susceptible to the two extracts.

### 2.4. Biofilm Eradication Activity of S. hillii Extracts

**Figure 1:**
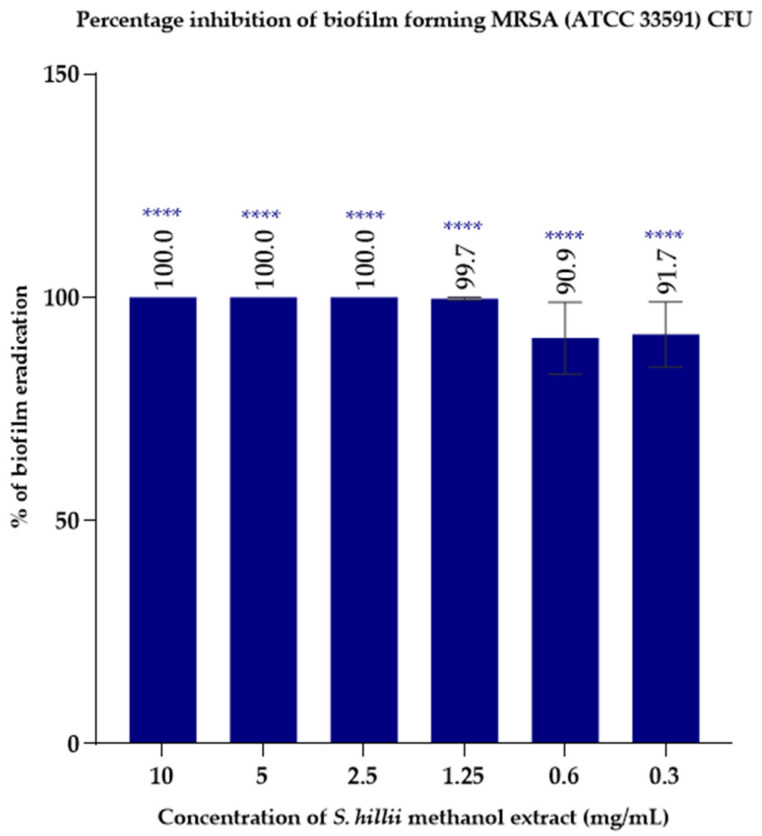
Biofilm eradication activity of *S. hillii* methanol extract against biofilm-forming MRSA (ATCC 33591) strain. The minimum biofilm eradication concentration (MBEC) of the *S. hillii* methanolic extract for MRSA (ATCC 33591) was 2.5 mg/mL. The average percentage inhibition of colony forming units (CFU) was measured by direct enumeration of viable MRSA colony forming units using ImageJ software and are expressed as the percentage mean of triplicates (n = 3) ± standard error (SEM). MRSA: methicillin-resistant *Staphylococcus aureus*. Significance levels **** *p* < 0.0001 compared to untreated control.

Based on antibacterial data obtained from previous experiments in which the methanol extract was most efficacious, its biofilm eradication ability was assessed. The average percentage reduction of colony forming units (CFU) was measured at different concentrations ranging between 0.3 and 10 mg/mL. The minimum biofilm eradication concentration (MBEC) of MRSA (ATCC 33591) when treated with the *S. hillii*-derived methanolic extract was 2.5 mg/mL (Figure 1).

### 2.5. Antimicrobial Activity of Primary Column Fractions of S. hillii-Derived Methanolic Extract

**Figure 2:**
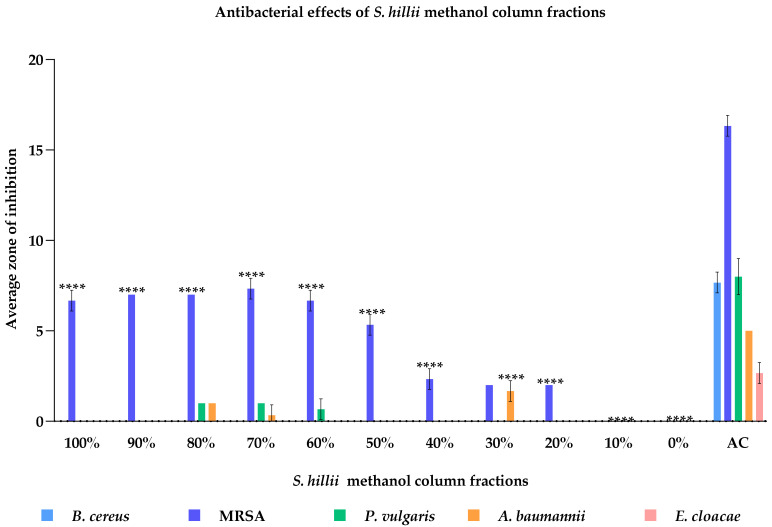
Antibacterial effects of *S. hillii* column fractions against *B. cereus*, MRSA, *P. vulgaris*, *A. baumannii*, and *E. cloacae* at 10 mg/mL. *B. cereus* (ATCC 14579) was resistant to all methanol fractions at the tested concentration. 20–100% fractions showed antibacterial effects against MRSA (ATCC 33591). The 30% fraction elicited the highest antibacterial effects against *A. baumannii* (ATCC 19606). However, the antibacterial activities of all the fractions were significantly low compared to the antibiotic standards (*p* < 0.0001). Eleven fractions were tested against five bacterial species, which were shown to be susceptible to the *S. hillii* methanol extract in the preliminary antibacterial screening. ZOI (zone of inhibition) was measured using the radius from the edge of the well to the edge of the clear zone (mm) and are expressed as the mean of triplicates ± standard error (SEM). Significance levels **** *p* < 0.0001 compared to antibiotic control (AC). MRSA: methicillin-resistant *Staphylococcus aureus*. AC: antibiotic control.

Due to antimicrobial activity, the methanolic extract was fractionated by column chromatography using a dichloromethane:methanol gradient system. This resulted in 11 fractions that were subsequently screened against *B. cereus* (ATCC 14579), MRSA (ATCC 33591), *P. vulgaris* (ATCC 7002), *A. baumannii* (ATCC 19606), and *E. cloacae* (ATCC 13047) at 50 mg/mL via the WDA (Figure 2). Both 70% and 80% methanolic fractions were highly effective against the tested bacteria; however, the overall efficacy was significantly lower compared to the antibiotic standard. *B. cereus* (ATCC 14579) was found to be resistant to all methanol fractions at 50 mg/mL. Fractions containing 30–80% methanol were shown to inhibit the growth of MRSA (ATCC 33591), *P. vulgaris* (ATCC 7002), *A. baumannii* (ATCC 19606), and *E. cloacae* (ATCC 13047), although at differing degrees. Moreover, the 90–100% methanolic fractions inhibited the growth of MRSA (ATCC 33591), while the 30% fraction inhibited *A. baumannii* (ATCC 19606). However, the antimicrobial activity of the respective standard antibiotic control was significantly higher compared to all *S. hillii*-derived methanol fractions at the concentration tested.

### 2.6. Isolation of Compounds from Primary Methanolic Fractions

**Figure 3:**
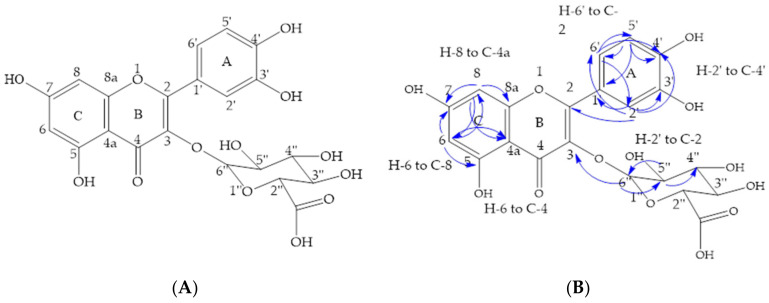
(**A**) Chemical structure of quercetin-3-O-β-D-glucuronide isolated from combined 70% and 80% fraction of the *S. hillii* methanol extract. (**B**) Key correlations of the heteronuclear multiple bond correlation (HMBC) of quercetin-3-O-β-D-glucuronide (images: ChemDraw 18.1).

**Figure 4:**
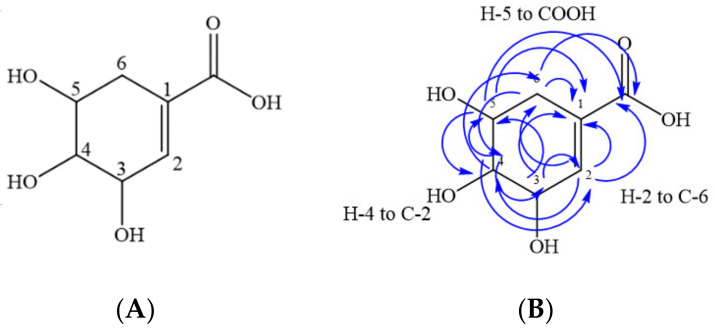
(**A**) Chemical structure of shikimic acid isolated from 30% fraction of the *S. hillii* methanol extract. (**B**) Key correlations of the heteronuclear multiple bond correlation (HMBC) of shikimic acid (images: ChemDraw 18.1).

Among the primary 30%, 70%, and 80% column fractions, all demonstrated antimicrobial activity against bacterial strains that are renowned for being highly resistant to antibiotics. As such, they were further fractionated using preparative HPLC. Due to similarities in HPLC chromatogram profiles and antibacterial activity, the 80% and 70% fractions were pooled. Quercetin-3-O-β-D-glucuronide was isolated from the pooled fraction (Figure 3), whereas shikimic acid was isolated from the 30% methanolic fraction (Figure 4).

Eleven milligrams of quercetin-3-O-β-D-glucuronide was isolated (Figure 3A), with structure elucidation based on the analyses of ^1^H and ^13^C NMR (Table 4), correlated spectroscopy (COSY), heteronuclear multiple bond correlation (HMBC), and heteronuclear single quantum coherence (HSQC) NMR experiments. The NMR spectra are provided in the Supplementary Materials File 1. The aromatic region of the ^1^H-NMR spectrum of the specified fraction revealed a doublet for two protons at 6.74 and 7.50 ppm, which were assigned to H-5′ and H-6′ hydrogens of ring A, respectively, whereas the singlet at 7.58 ppm was assigned to the H-2′ hydrogen of ring A. A set of doublets at 6.10 and 6.29 ppm in the aromatic region were assigned to H-6 and H-8 hydrogens of ring C, respectively. In the aliphatic region of the spectrum, three triplets at 3.35, 3.42, and 3.47 ppm were assigned to H-4′′, H-5′′, and H-3′′ of the glycosylated sugar moiety, respectively. The key anomeric proton appeared at 5.23 ppm. The ^13^C NMR spectrum confirmed that there were 15 carbons in the molecule. Further characterization by HSQC- and HMBC-2D-NMR revealed carbon-hydrogen (C-H) single bond correlations and multiple bond correlations for the aromatic region. Key correlations from HSQC revealed that H-2′ (7.58), H-5′ (6.74), and H-6′ (7.50) were attached to C-2′ (115.9), C-5′ (114.6), and C-6′ (121.9) of ring A; H-6 (6.10) and H-8 (6.29) to C-6 (98.5) and C-8 (93.3) of ring C; and H-2′′ (5.90), H-3′′ (5.90), H-4′′ (5.90), and H-5′′ (5.90) to C-2′′ (76.2), C-3′′ (71.5), C-4′′ (76.2), and C-5′′ (74.0) of the sugar moiety, respectively.

The key HMBC three-bond correlations were from H-6 to C-5 (161.0), C-7 (164.6), C-8 (93.3), and C-4a (104.2); H-8 to C-6 (98.5), C-7 (164.6), C-8a (157.0), and C-4a (104.2); H-2′ to C-2 (157.6), C-1′ (121.4), C-3′ (144.6), C-4′ (148.5), and C-6′ (121.9); H-5′ to C-1′ (121.4), C-3′ (144.6), C-4′ (148.5), and C-6′ (121.9); and H-6′ to C-2 (157.6), C-2′ (115.9), C-4′ (148.5), and C-5′ (114.6) (Figure 3B). A key correlation was observed from H-6′ to C-3 (157.6), indicating that C-3 was glycosylated. In addition, the obtained proton NMR values are consistent with the published literature [14]. These HMBC correlations verified the structure as quercetin-3-O-β-D-glucuronide. The ESI–MS produced a mass ion peak in negative mode at *m/z* 477.0677, further confirming the structure.

A total of 586 mg of shikimic acid was isolated (Figure 4A), with the structure determined using the same analytical techniques previously used to identify quercetin (Table 5). The NMR spectra are provided in the Supplementary Materials File 1. In the ^1^H spectrum, singlets at 6.58 and 4.20 ppm were assigned to H-2 and H-3 protons, and multiplets at 3.52–3.54 and 3.81–3.84 ppm were assigned to H-4 and H-5 protons of the compound, respectively. The methylene protons at H-6 of shikimic acid appeared as doublets at 1.99 and 2.40 ppm. The ^13^C NMR spectrum confirmed that seven carbons are contained within the molecule. Further characterization by HSQC- and HMBC-2DNMR revealed carbon-hydrogen (C-H) single bond correlations and multiple bond correlations for the assigned structure. Key correlations from HSQC revealed that H-2 (6.58), H-3 (4.20), H-4 (3.52–3.54), H-5 (3.81–3.84), H-6a (2.40), and H-6b (1.99) were attached to C-2 (138.4), C-3 (65.9), C-4 (70.9), C-5 (67.2), C-6a (30.6), and C-6b (30.6), respectively.

The key HMBC correlations were from H-2 to C-1 (129.5), C-4 (70.9), C-6 (30.6), and COOH (168.8); H-3 to C-1 (129.5), C-2 (138.4), and C-5 (67.2); H-4 to C-2 (138.4), C-3 (65.9), C-5 (67.2), and C-6 (30.6); H-5 to C-1 (129.5), C-3 (65.9), C-4 (70.9), and COOH (168.8); H-6a and H-6b to C-1 (129.5), C-2 (138.4), C-4 (70.9), and C-5 (67.2) (Figure 4B). HMBC correlations confirmed the structure as shikimic acid, which was further supported as a mass ion peak in negative mode at *m/z* 174.05 was observed during the ESI–MS analysis. In addition, the obtained proton NMR values are consistent with the published literature [15,16].

### 2.7. Antimicrobial Activity of Isolated Compounds from the S. hillii Methanolic Extract

Quercetin-3-O-β-D-glucuronide and shikimic acid were screened against eight bacteria, including several ESKAPE pathogens. MIC values ranged from 0.78 to 200 µg/mL (Table 6). Both compounds exhibited antibacterial effects against the target bacteria, although at varying concentrations. Shikimic acid inhibited the growth of *E. faecalis* (QUT 1105), *E. cloacae* (ATCC 13047), and *P. aeruginosa* (ATCC 27853) at 200 µg/mL. Similarly, quercetin-3-O-β-D-glucuronide was effective against *P. vulgaris* (ATCC 6380), *P. aeruginosa* (ATCC 27853), and *E. cloacae* (ATCC 13047) at the same concentration, with the compound exerting potent antimicrobial activity against *E. faecalis* (QUT 1105) (MIC-0.78 µg/mL). In general, *B. cereus* (ATCC 14579), MRSA (ATCC 33591), *A. baumannii* (ATCC 19606), and *K. pneumoniae* (ATCC 27736) were the most resistant to the isolated compounds.

### 2.8. Predicted ADME Properties of the Isolated Compounds

Given the potency of quercetin-3-O-β-D-glucuronide against *E. faecalis* (QUT 1105)*,* in silico absorption, distribution, metabolism, and excretion (ADME) properties of the compound were determined (Table 7). Molecular weight, number of hydrogen bond acceptors and donors, molar refractivity, total polar surface area, and logP are the most important physicochemical properties that are taken into account during drug development. Quercetin-3-O-β-D-glucuronide follows the recommended limit; however, the number of hydrogen bond donors and acceptors were shown to be outside the limit. Further, gastrointestinal (GI) absorption of the compound was also below the lower limit. According to the computationally derived properties, quercetin-3-O-β-D-glucuronide does not inhibit multiple cytochrome P450 enzymes, a group of enzymes essential for the metabolism of drugs.

## 3. Discussion

The genus *Syncarpia* has been used by the Yaegl community (New South Wales, Australia) for various medicinal purposes for many years [5]. Records concerning the use of *S. hillii* claim that the plant has the ability to heal sores and chronic ulcers, and the sap and ash from the leaves of related species, e.g., *S. glomulifera*, are said to have antiseptic properties [5,9]. Extracts derived from *S. hillii* showed antimicrobial activity against 80% of the bacterial strains screened, including pathogens that cause skin infections such as *S. aureus*. Ethanol and methanol extracts of *S. hillii* produced significantly higher activity against the bacterial species tested compared to other primary extracts. Further, both extracts demonstrated the highest inhibitory effects against Gram-positive *Staphylococcal* spp. compared to the remaining bacteria. Overall, Gram-negative bacteria were found to be impervious to *S. hillii* extracts regardless of the solvent used. Moreover, of the eight Gram-negative bacteria screened, only *P. vulgaris* (ATCC 7002), *P. mirabilis* (ATCC 6380), *A. baumannii* (ATCC 19606), and *E. cloacae* (ATCC 13047) were shown to be susceptible to the extracts; however, the effect was minimal. This result suggests that the compounds contained within the extracts, irrespective of solvent, are able to penetrate the peptidoglycan cell wall of Gram-positive bacteria and subsequently exert their bactericidal effects. Unlike Gram-negative bacteria, which, in addition to the same murein cell wall, also contain an outer membrane, it appears to be highly resistant to *S. hillii*-derived compounds.

Akter et al., tested the leaves of *S. glomulifera* against Gram-positive bacteria that included MSSA, MRSA, wild multi-drug-resistant *S. aureus*, and Gram-negative strains, *P. aeruginosa* and *E. coli* [5]. Following a similar extraction process as carried out in this study, the authors demonstrated that the ethanol extract of *S. glomulifera* was bactericidal against all *Staphylococcus* species screened [5]. These results mirrored our own to some extent; however, MIC values of the *S. glomulifera* extracts against the three *S. aureus* species were found to be 160 times more effective (7.81 μg/mL) compared to those (1.25 mg/mL) generated in our study. This suggests that even though both plants are members of the same family and share the same genus, *S. glomulifera* contains compounds that, overall, either have higher antibacterial potency, or the concentration of the bioactive(s) within the plant is much greater compared to *S. hillii*. In addition, morphogenetic, ontogenic, and environmental factors can also influence the biosynthesis and accumulation of plant compounds or secondary metabolites [17].

The MBC is the lowest concentration required to completely kill a pathogen. MBC values demonstrated in our study were much higher compared to the MIC values, thereby confirming that the extracts are bactericidal at a higher concentration and bacteriostatic at lower concentrations. The ratio of MBC to MIC provides information regarding the degree of bactericidal activity, i.e., a narrow ratio suggests higher activity, whereas a wide ratio is indicative of poor activity [18]. However, the bactericidal effect of a treatment is not only dependent upon antibiotic concentration, but also on the bacterial species being targeted, the inoculum density of the bacteria, as well as environmental conditions, such as pH and protein concentration [18]. The MBC values for the Gram-positive strains ranged between 5 and 20 mg/mL for the ethanol and methanol extracts, while the aqueous and isopropanol extracts were 10–20 mg/mL. Interestingly, the MBC value of the ethanol extract against various *Staphylococcal* strains was slightly lower than that of the methanol extract in contrast to the MIC values. This might be due to differences in detection methods used to determine the MIC and MBC values, as it has been reported that INT is reduced by different microorganisms at varying rates [19].

*P. aeruginosa* and *S. aureus* are reported to have the greatest number of biofilm-producing strains [20], although *E. coli* also generates biofilms 24–48 h post-infection [21,22]. Globally, 60% of all infected chronic wounds contain a biofilm [23,24]. Sessile bacterial cells in biofilms are considered highly resistant to heat and desiccation, acids, and antibiotics because of the high density of their extracellular polymeric structure [25]. A recent study showed that the resistance of biofilm-protected sessile bacterial cells are usually 10 to 1000 times higher compared to its planktonic counterparts [26]. The biofilm formation ability of MRSA has been identified as one of the major reasons behind its multidrug resistance [26]. MRSA exhibits major resistance to all available penicillins and most β-lactam drugs [26]. In our study, we aimed to determine whether the methanol extract obtained from *S. hillii* had the ability to impede biofilm formation as it had the greatest antibacterial activity of the plant fractions tested. The antibiofilm assay protocol was based on a direct enumeration of bacterial colony forming units produced by biofilm-forming MRSA (ATCC 33591). The bacterium was treated with the *S. hillii*-derived methanolic extract over a concentration gradient. Colony counts of the treated MRSA were compared against an untreated control, and then the average percentage inhibition of CFUs was calculated. The MBEC of MRSA for the *S. hillii* methanol extract was found to be 2.5 mg/mL. According to our study, it was evident that the concentration of the *S. hillii* methanolic extract needed to eradicate sessile MRSA cells is approximately fourfold higher compared with planktonic MRSA cells. However, due to the methodological differences used to detect the MIC and MBEC values, it is difficult to compare the magnitude of resistance of sessile bacteria in biofilms. There are several methods that are used to detect different parameters related to MRSA biofilm formation, although none are without drawbacks. For example, crystal violet and safranin assays are used to detect biofilm biomass, whilst fluorophores such as SYTO9 and propidium iodide (PI), based on live/dead cells, detect the metabolic rate of sessile bacteria within biofilms, and scanning electron microscope imaging is able to capture sessile bacteria trapped in a polysaccharide biofilm [27].

Based on the high efficacy of the *S. hillii* methanol extract, it was subjected to further fractionation and compound isolation. Of the 11 primary fractions resultant from the extract, eight were effective against at least one of the bacterial strains screened. Overall, methanol 70% and 80% fractions were the most efficacious against the bacterial strains tested, including MRSA (ATCC 33591). The 30% methanolic fraction had a bactericidal effect on *A. baumannii*, although it was minimally effective against Gram-positive bacteria. *A. baumannii* is an important opportunistic pathogen that has caused global outbreaks of nosocomial infections [28]. Therefore, the 30%, 70%, and 80% methanol fractions were subjected to further testing for potential compound isolation. Due to the similarities of the HPLC chromatogram profiles and antibacterial activity observed, the 80% and 70% methanol fractions were pooled. Quercetin-3-O-β-D-glucuronide was subsequently isolated from the combined fraction, while shikimic acid was isolated from the 30% methanol fraction. Of both compounds, quercetin-3-O-β-D-glucuronide was shown to be highly selective as it inhibited *E. faecalis* with a MIC value of 0.78 μg/mL. Further, it impeded the growth of *P. vulgaris* (ATCC 6380), *E. cloacae* (ATCC 13047), and *P. aeruginosa* (ATCC 27853) at 200 μg/mL. Shikimic acid also inhibited the growth of *E. faecalis*, *E. cloacae*, and *P. aeruginosa* at 200 μg/mL. However, at a concentration of 10 mg/mL, *E. faecalis* (QUT code 1105), *P. vulgaris* (QUT code 1105), *E. cloacae* (ATCC 13047), and *P. aeruginosa* (ATCC 27853) were impervious to the methanolic extract. The reason for this contrasting result may be due to the specific compounds responsible for the activity being at much lower concentrations in the extract during the initial screening.

Interestingly, MRSA (ATCC 33591), the most susceptible bacterial species to the *S. hillii* methanol extract, was not inhibited by either of the isolated compounds at 200 μg/mL. This finding suggests that the synergism between compounds in a crude extract may result in loss of activity during the bioassay-guided fractionation process [29]. Two other studies determined the MIC value of quercetin against MRSA (ATCC 33591) [30] and a MRSA clinical isolate (ATCC 43300) [31] to be 300 μg/mL and 500 μg/mL, respectively. Based on these reported results, the concentrations used in our study were too low to inhibit the growth of MRSA. Nonetheless, to verify this, future MIC assays should be performed at concentrations 2.5-fold higher. However, the amount of quercetin-3-O-β-D-glucuronide obtained in our study was relatively low, thereby severely limiting the concentration at which the compound could be tested, given its overall yield. Shikimic acid has also been reported to possess antibacterial activity against *S. aureus* at 2.5 mg/mL as it has the ability to damage cell membrane permeability [32]. This is relatively a very high concentration compared to what was used in our study. Further, the *S. aureus* strain (ATCC 6538) that Bai and colleagues [32] used is different to the bacterium we screened, hence making a direct comparison impossible.

*Enterococci* are important healthcare-associated pathogens that cause nosocomial infections [33,34] including urinary tract, intra-abdominal, pelvic and soft tissue infections [33]. Moreover, *Enterococci* are the second most common cause of bacteremia [33]. However, treatment of *E. faecalis* is challenging as the bacterium has developed intrinsic and acquired resistance to many antibiotics, including vancomycin, ampicillin, aminoglycosides, β-lactams, macrolides, cephalosporin, tetracycline, and fluoroquinolones [33,35]. Interestingly, quercetin-3-O-β-D-glucuronide tested in our study showed excellent inhibitory activity against *E. faecalis* (QUT code 1105) and has the potential for future drug development. As such, the physicochemical properties of quercetin-3-O-β-D-glucuronide were calculated using the SwissADME online tool. Some of the physicochemical properties such as number of hydrogen donor and acceptor bonds were outside the limit. This result is due to the presence of hydroxyl and phenolic groups contained within the structure. Further, 136 molecules are reported to have structural similarities (95–98%) to quercetin-3-O-β-D-glucuronide within Scifinder. Hence, these molecules could be used initially to develop the structure–activity relationships of the compounds and to subsequently identify more potent analogs of quercetin-3-O-β-D-glucuronide, which can then be further optimized using classical synthetic medicinal chemistry techniques.

## 4. Materials and Methods

### 4.1. Collection and Preparation of the Primary Leaf Extracts

*S. hillii* mature leaves, confirmed by a botanist at Narangba nursery, Brisbane Queensland, were purchased from Daley Nurseries (Geneva, New South Wales, Australia), collected, dried, and ground into a fine powder using an herb grinder. The powder was then individually extracted using five different solvents of varying polarities. Aqueous, methanol (ThermoFisher Scientific, Massachusetts, MA, USA), and ethanol (ThermoFisher Scientific, Massachusetts, MA, USA) extracts of *S. hillii* were re-dissolved in milli-Q water (arium™ pro, Sartorius, Germany), while the isopropanol (Merck Germany) and hexane (Merck, Germany) extracts were redissolved in 10% isopropanol in milli-Q water to obtain a final extract concentration of 100 mg/mL.

### 4.2. Bacterial Cultures

Bacterial cultures were either purchased from the ATCC via In Vitro Technologies (Melbourne, Australia) or obtained from Dr. Juliana Chiruta (QUT, Brisbane, Australia). Methicillin-resistant *S. aureus* (MRSA) (ATCC 33591 and clinical isolate QUT code 1113), MSSA (NCTC 6571), *B. cereus* (ATCC 14579), *K. pneumoniae* (ATCC 27736), *E. coli* (ATCC 25922), *P. aeruginosa* (ATCC 27853), *B. subtilis* (QUT code 0535), *S. epidermidis* (QUT code 0613), *P. vulgaris* (ATCC 6380), *P. mirabilis* (ATCC 7002), *E. faecalis* (QUT code 1105), *E. faecium* (QUT code 1101), multi-drug resistant *A. baumannii* (ATCC 19606), *E. gallinarum* (ATCC 49573), *E. casseliflavus* (ATCC 25788), *E. aerogenes* (ATCC 13048), *E. cloacae* (ATCC 13047), and *S. saprophyticus* (QUT code 0703) were used. Individual strains were streaked onto nutrient agar plates and incubated at 37 °C for 24 h, excluding *B. subtilis*, which was incubated at 28 °C for 24 h. Culture plates were stored at 2–8 °C until required.

### 4.3. Well Diffusion Assay (WDA)

Before each antimicrobial test, a fresh subculture in Mueller–Hinton (MH) agar (Oxoid Ltd., Thebarton, Australia) was used to make the various bacterial saline suspensions. Briefly, one colony from the fresh bacterial subculture was suspended into 1 mL of sterile 0.9% w/v saline suspension and adjusted to equate to a 0.5 McFarland standard. MH agar plates (ThermoFisher Scientific, Victoria, Australia) were then inoculated evenly with 200 μL of an individual bacterial species using a sterile disposable spreader (SARSTEDT AG & Co., Nümbrecht, Germany).

Wells were aseptically punched into the agar using a 6 mm biopsy punch (ThermoFisher Scientific, Victoria, Australia) and filled with 80 μL of extracts at a concentration of 100 mg/mL. Standard antibiotic discs (Oxoid Ltd., Hampshire, UK) were used as positive controls, whereby trimethoprim (1.25 μg) + sulfamethoxazole (23.75 μg) acted as the control for both MRSA isolates (QUT 1113 and ATCC 33591), *S. epidermidis* (QUT 0613), *S. saprophyticus* (QUT 0703), *P. vulgaris* (ATCC 6380), and *P. mirabilis* (ATCC 7002); penicillin G (10 μg) was used for MSSA (NCTC 6571); erythromycin (15 μg) was used for *B. cereus* (ATCC 14579) and *B. subtilis* (QUT 0535); gentamicin (10 μg) was used for *K. pneumoniae* (ATCC 27736), *E. coli* (ATCC 25922), *P. aeruginosa* (ATCC 27853), *A. baumannii* (ATCC 19606), *E. aerogenes* (ATCC 13048), and *E. clocae* (ATCC 13047). Teicoplanin (30 μg) was used for *E. faecalis* (QUT code 1105), *E. casseliflavus* (ATCC 25788), and *E. gallinarum* (ATCC 49573), while linezolid (30 μg) was used for *E. faecium* (QUT code 1101). Plates were then incubated at 37 °C for 24 h excluding *B. subtilis* (QUT 0535), which was incubated at 28 °C, and the subsequent ZOI (denoted as the radius from the edge of the well to outer margin of clear zone) was measured (mm).

### 4.4. Minimum Inhibitory Concentration (MIC)

MIC values of the plant extracts were determined using sterile 96-well plates (ThermoFisher Scientific, Victoria, Australia). An amount of 50 μL of each bacterial suspension (0.5 McFarland standard) was added to each well. Cells with 50 μL of MH broth and the corresponding bacterial suspension was used as the positive control. Conversely, bacterial suspensions with 50 μL of 10 mg/mL of corresponding antibiotic were served as the antibiotic control. A diluted series of 50 μL plant extracts in MH broth (0.02–10 mg/mL) was added to the treatment wells. Separately, 50 μL of extract (0.02–10 mg/mL) in MH broth was added to serve as the background control of the extracts.

The 96-well plates were subsequently sealed and incubated overnight at 37 °C. Next, 40 μL of INT dye (2-p-iodophenyl-3-pnitrophenyl-5-phenyl tetrazolium chloride) (Sigma-Aldrich, St. Louis, MO, USA) at 0.125 mg/mL was added to each well and incubated for 1 h at 37 °C. Finally, the absorbance was detected using POLARstar Omega plate reader (BMG Labtech Pty. Ltd., Ortenberg, Germany), at a wavelength of 550 nm. The lowest concentration of plant extract at which the color changed from yellow to pink was considered the MIC value.

### 4.5. Minimum Bactericidal Concentration (MBC)

Each bacterial species (0.5 McFarland standard) was diluted 1:100 in MH broth and incubated overnight at 37 °C with shaking. A 10× dilution series (2.5–20 mg/mL) of each extract was made in MH broth. An amount of 90 μL of the diluted bacterial suspension was individually added to each well. In addition, 10 μL of the extracts was then added to each corresponding treatment well, while 10 μL of MH broth was used as the positive control, and 10 μL of the antibiotic (10 mg/mL) served as the antibiotic control to each corresponding bacterial strain. Plates were sealed and incubated overnight at 37 °C. Then, 2 μL from each corresponding well was inoculated onto a fresh MH agar plate with a separate plate for each bacterial species. Plates were sealed and subsequently incubated overnight at 37 °C. The lowest concentration, where zero bacterial growth was observed, determined the MBC.

### 4.6. Direct Enumeration Method for Biofilm Eradication

Biofilm forming MRSA (ATCC 33591) was grown overnight at 37 °C in nutrient broth media (Sigma-Aldrich, New South Wales, Australia) and then diluted 1:100 in fresh M63 minimal medium supplemented with magnesium sulphate (Sigma-Aldrich, New South Wales, Australia) and arginine (Sigma-Aldrich, New South Wales, Australia). An amount of 100 μL of the diluted culture in M63 (Mg/Arg) was added to wells of the flat-bottomed 96-well microplate strips (Corning, Massachusetts, MA, USA; catalogue no: CORN9102), which was incubated for 48 h at 37 °C under static aerobic conditions. After incubation, the spent supernatant containing planktonic cells were removed using a multichannel pipette (Nichipet EXII, Taufkirchen, Germany) and 90 μL of M63 (Mg/Arg) was added to all wells. Then, 10 μL of each 10X concentrated plant extract was added to achieve the desired final concentrations (0.3–10 mg/mL). An amount of 10 μL of sterile water was added to the untreated control. The plate was incubated overnight at 37 °C. The contents from each preparation were aspirated and rinsed three times with 200 μL of PBS (Astral Scientific, Taren Point, New South Wales, Australia). Each labeled well was separated from the microtiter plate and inserted to separate 8 mL sterile tubes containing 1.9 mL of PBS and capped after addition. The contents of each tube were sonicated for 8 s at 40% power. The resulting suspensions were inoculated on MH agar medium and viable cell counts were performed on the resulting bacterial colonies. It was assumed that the colonies formed from sessile bacteria that were trapped onto biofilms. Direct enumeration of colonies was performed using ImageJ software [36]. The following formula was used for the percentage inhibition calculation. Percentage Inhibition = (CFU in untreated sample—CFU in the treated sample)/CFU in the untreated sample * 100 (CFU: colony forming units).

### 4.7. Antimicrobial Activity of S. hillii Methanol Extract Column Fractions

Antimicrobial activity of the *S. hillii* methanol extract column fractions was determined using the WDA method. Based on the preliminary antimicrobial activity, five bacterial species including Gram-positive MRSA (ATCC 33591) and *B. cereus* (ATCC 14579), as well as Gram-negative *P. vulgaris* (ATCC 7002), *A. baumannii* (ATCC 19606), and *E. cloacae* (ATCC 13047), were selected to test the antimicrobial potential of 11 column fractions of *S. hillii* methanol extract at 50 mg/mL. Column fractions were re-dissolved in milli-Q water (arium™ pro, Sartorius, Germany), or 10% isopropanol in milli-Q water depending upon the polarity of each fraction.

### 4.8. Isolation of Bioactive Compounds from S. hillii Leaf Extracts

Bioactive compounds were isolated and identified following specific and distinctive steps, including bioassay-guided fractionation and isolation of the compounds from the primary methanol extract of *S. hillii*. Primary fractions were eluted with a solvent gradient consisting of 600 mL volume fractions with 10% (*v*/*v*) increments in each progressive step with dichloromethane (DCM) (Thermo Fisher Scientific, Scoresby, Victoria, Australia) and methanol using a glass column containing 50 g of silica gel (Merck, Darmstadt, Germany) 60 Å conditioned with hexane. Testing for bioactivity was carried out at each primary fraction processing step. Active primary fractions collected from *S. hillii* methanol extracts were separated by preparative and analytical HPLC C18 columns. The mobile phase consisted of (A) 0.05% *v*/*v* formic acid in milli-Q water and (B) methanol. Compound structures were identified by ^1^H, ^13^C, and 2D NMR spectra (COSY, HSQC, and HMBC) recorded at 600 MHz on an Ascend^TM^ 600 spectrometer (Bruker, Thebarton, Australia). TopSpin^TM^ 4.0.6 software was used to analyze the NMR data. Finally, samples were analyzed on positive and negative ion modes with a range of *m*/*z* 100–800, at a scan rate of 0.5 Hz on a LTQ XLTM ion trap mass spectrometer (Thermo Scientific, Victoria, Australia) to determine and confirm the molecular weights of the structures.

### 4.9. Antimicrobial Activity of the Isolated Compounds

The antimicrobial activity and the MIC values of the isolated compounds were determined using the same semiquantitative method described in Section 4.4. Isolated compounds were initially dissolved in milli-Q water and diluted in MH broth. Four-fold dilution series consisting of final concentrations ranging between 0.78 and 200 µg/mL were tested against eight bacterial species, including Gram-positive, Gram-negative, and several ESKAPE pathogens.

### 4.10. Statistical Analysis

Statistical analyses were performed using GraphPad Prism 8.4.3 software. Triplicate samples were assayed in each technical experiment and replicated three times. The comparison among multiple groups was performed by two-way analysis of variance (ANOVA) followed by Dunnett’s multiple comparisons test. The statistically significant levels were set at *p* < 0.05 (*), *p* < 0.01 (**), *p* < 0.001 (***), and *p* < 0.0001 (****).

## 5. Conclusions

This is the first project to phytochemically investigate the pathological significance of *S. hillii* leaf extracts and cognate bioactive compounds. Overall, *Staphylococcal* species had the greatest susceptibility to methanolic extracts derived from the leaves of *S. hillii* as it produced the greatest antibacterial effects, including the impediment of biofilm formation. Two bioactive compounds were isolated and identified as quercetin-3-O-β-D-glucuronide and shikimic acid from the *S. hillii* methanol extract and demonstrated improved antibacterial effects against *E. faecalis* (QUT code 1105), *P. vulgaris* (ATCC 6380), *E. cloacae* (ATCC 13047), and *P. aeruginosa* (ATCC 27853). Consequently, this project has scientifically confirmed the potential antibacterial properties of *S. hillii* leaves, used traditionally to heal skin infections.

## Figures and Tables

**Table 1:** Antibacterial effects of *S. hillii* primary leaf extracts.

Bacterial Strain	Antibiotic Standard ZOI (mm)	Average ZOI (mm) for *S. hillii* Extracts (100 mg/mL)				
		Aqueous	Methanol	Ethanol	Isopropanol	Hexane
Gram-positive bacteria						
*Bacillus cereus* (ATCC 14579)	7.00 ± 0.00	3.56 ± 0.50	5.00 ± 0.00	5.28 ± 0.42	3.22 ± 0.63	1.56 ± 0.68
MSSA (NCTC 6571)	11.33 ± 1.89	4.44 ± 0.50	5.89 ± 0.54	6.00 ± 0.82	4.22 ± 0.63	3.89 ± 0.74
MRSA (QUT 1113)	8.33 ± 0.24	6.44 ± 0.50	7.33 ± 0.45	6.67 ± 0.47	5.78 ± 0.92	3.56 ± 0.50
MRSA (ATCC 33591)	7.00 ± 0.00	6.56 ± 0.83	6.33 ± 0.45	5.89 ± 0.57	5.78 ± 0.92	2.89 ± 0.87
*Bacillus subtilis* (QUT 0535)	9.00 ± 0.00	NA	4.33 ± 0.45	4.00 ± 0.00	1.89 ± 0.57	1.67 ± 0.47
*Staphylococcus epidermidis* (QUT 0613)	9.00 ± 0.00	3.67 ± 0.47	5.67 ± 0.45	5.17 ± 0.33	2.67 ± 0.67	0.56 ± 0.50
*Enterococcus faecalis* (QUT code 1105)	3.00 ± 0.00	NA	2.33 ± 0.45	2.33 ± 0.47	NA	1.89 ± 0.31
*Enterococcus faecium* (QUT code 1101)	6.00 ± 0.00	NA	2.33 ± 0.45	2.78 ± 0.42	2.00 ± 0.82	2.33 ± 0.47
*Enterococcus gallinarum* (ATCC 49573)	4.00 ± 0.00	0.89 ± 0.74	1.33 ± 0.45	2.67 ± 0.67	NA	2.67 ± 0.47
*Enterococcus casseliflavus* (ATCC 25788)	4.00 ± 0.00	NA	2.00 ± 0.00	2.00 ± 0.67	NA	0.67 ± 0.47
*Staphylococcus saprophyticus* (QUT 0703)	8.00 ± 0.00	4.22 ± 0.42	5.00 ± 0.00	4.78 ± 0.42	3.78 ± 0.42	2.22 ± 0.42
Gram-negative bacteria						
*Klebsiella pneumoniae* (ATCC 27736)	3.00 ± 0.00	NA	NA	NA	NA	NA
*Pseudomonas aeruginosa* (ATCC 27853)	5.00 ± 0.00	NA	NA	NA	NA	NA
*Escherichia coli* (ATCC 25922)	6.00 ± 0.00	NA	NA	NA	NA	NA
*Proteus vulgaris* (ATCC 6380)	6.33 ± 0.47	NA	1.67 ± 0.45	1.44 ± 0.50	0.11 ± 0.31	NA
*Proteus mirabilis* (ATCC 7002)	6.00 ± 0.00	NA	2.00 ± 0.00	1.78 ± 0.42	NA	NA
*Acinetobacter baumannii* (ATCC 19606)	4.00 ± 0.00	NA	1.00 ± 0.00	0.89 ± 0.57	NA	NA
*Enterobacter aerogenes* (ATCC 13048)	3.00 ± 0.00	NA	NA	NA	NA	NA
*Enterobacter cloacae* (ATCC 13047)	3.00 ± 0.00	NA	0.44 ± 0.47	0.22 ±0.42	NA	NA

All *S. hillii* extracts were antibacterial against Gram-positive strains. *P. vulgaris*, *P. mirabilis*, *A. baumannii*, and *E. cloacae* were susceptible to methanol and ethanol extracts derived from *S. hillii.* Overall, the methanol and ethanol extracts were the most efficacious against the bacterial strains tested. ZOIs (zones of inhibition) were measured as the radius from the edge of the well to the edge of the clear zone (mm) and are expressed as the mean of triplicates ± standard error (SEM). SXT: trimethoprim + sulfamethoxazole. NA: no activity. Negative control: sterile milli-Q water or 10% isopropanol (0 ± 0.0 mm). MSSA: methicillin-sensitive *Staphylococcus aureus*. MRSA: methicillin-resistant *Staphylococcus aureus*. Standard antibiotic discs were used as positive controls, whereby trimethoprim (1.25 μg) + sulfamethoxazole (23.75 μg) acted as the control for both MRSA isolates, *S. epidermidis*, *S. saprophyticus*, *P. vulgaris*, and *P. mirabilis*; penicillin G (10 μg) for MSSA; erythromycin (15 μg) for *B. cereus* and *B. subtilis*; gentamicin (10 μg) for *K. pneumoniae*, *E. coli*, *P. aeruginosa*, *A. baumannii*, *E. aerogenes*, and *E. clocae*; teicoplanin (30 μg) for *E. faecalis*, *E. casseliflavus*, and *E. gallinarum*; and linezolid (30 μg) was used for *E. faecium*. Test performed in triplicate and repeated three times (n = 3).

**Table 2:** MIC of *S. hillii* methanol and ethanol against bacterial strains.

Bacterial Strain	Antibiotic Standard (µg/mL)	*S. hillii* Extracts (mg/mL)				
		Methanol	Ethanol	Aqueous	Isopropanol	Hexane
Gram-positive bacteria						
*Bacillus cereus* (ATCC 14579)	5.00	1.25	2.50	10.00	5.00	>10.00
MSSA (NCTC 6571)	5.00 *	1.25	1.25	5.00	5.00	>10.00
MRSA (QUT 1113)	5.00 *	0.63	1.25	2.50	2.50	>10.00
MRSA (ATCC 33591)	5.00 *	0.63	0.63	2.50	2.50	>10.00
*Bacillus subtilis* (QUT 0535)	2.50	5.00	5.00	ND	10.00	>10.00
*Staphylococcus epidermidis* (QUT 0613)	4.00 *	0.63	0.63	2.50	2.50	ND
*Staphylococcus saprophyticus* (QUT 0703)	30.00 *	1.25	1.25	5.00	ND	>10.00
Gram-negative bacteria						
*Proteus vulgaris* (ATCC 6380)	5.00	1.25	1.25	ND	ND	ND
*Proteus mirabilis* (ATCC 7002)	5.00	1.25	2.50	ND	ND	ND
*Acinetobacter baumannii* (ATCC 19606)	10.00 *	2.50	2.50	ND	ND	ND
*Enterobacter cloacae* (ATCC 13047)	20.00 *	1.25	1.25	ND	ND	ND

*Staphylococcal species* exhibited the greatest susceptibility to *S. hillii* extracts. MIC values were determined by the lack of INT reduction to INT formazan measured at 550 nm and are expressed as the mean of triplicates. ND: not determined. SXT: trimethoprim + sulfamethoxazole. MSSA: methicillin-sensitive *Staphylococcus aureus*. MRSA: methicillin-resistant *Staphylococcus aureus*. *: MIC values obtained from EUCAST (The European Committee on Antimicrobial Susceptibility Testing). Standard antibiotics were used as positive controls, whereby trimethoprim (1.25 μg) acted as the control for both MRSA isolates, *S. epidermidis*, *S. saprophyticus*, *P. vulgaris*, and *P. mirabilis*; penicillin G (10 μg) for MSSA; erythromycin (15 μg) for *B. cereus* and *B. subtilis*; gentamicin (10 μg) for *K. pneumoniae*, *A. baumannii*, and *E. clocae*. Test performed in triplicate and repeated three times (n = 3).

**Table 3:** MBC of *S. hillii* leaf extracts on screened bacterial strains.

Bacterial Strain	Tested Concentration of the Standard Antibiotic (mg/mL)	MBC of *S. hillii* Extracts (mg/mL)	
		Methanol	Ethanol
Gram-positive bacteria			
*Bacillus cereus* (ATCC 14579)	1.0	15.0	20.0
MSSA (NCTC 6571)	1.0	5.0	5.0
MRSA (ATCC 33591)	1.0	7.5	7.5
MRSA (QUT 1113)	1.0	7.5	5.0
*Bacillus subtilis* (QUT 0535)	1.0	15.0	15.0
*Staphylococcus epidermidis* (QUT 0613)	1.0	5.0	5.0
*Enterococcus faecalis* (QUT 1105)	1.0	>20.0	>20.0
*Enterococcus faecium* (QUT 1101)	1.0	>20.0	>20.0
*Enterococcus gallinarum* (ATCC 13048)	1.0	>20.0	>20.0
*Enterococcus casseliflavus* (ATCC 25788)	1.0	>20.0	>20.0
*Staphylococcus saprophyticus* (QUT 0703)	1.0	5.0	5.0
Gram-negative Bacteria			
*Proteus vulgaris* (ATCC 7002)	1.0	>20.0	>20.0
*Proteus mirabilis* (ATCC 6380)	1.0	>20.0	>20.0
*Acinetobacter baumannii* (ATCC 19606)	1.0	>20.0	>20.0
*Enterobacter cloacae* (ATCC 13047)	1.0	>20.0	>20.0

*Staphylococcal* species exhibited the greatest susceptibility to all the *S. hillii* extracts. Gram-negative strains exhibited resistance to methanol and ethanol *S. hillii* extracts at 20.0 mg/mL. MBC values were defined as the lowest concentration that ceased bacterial growth on the Mueller–Hinton agar/tryptic soy agar and are expressed as the mean of triplicates. SXT: trimethoprim + sulfamethoxazole. MSSA: methicillin-sensitive *Staphylococcus aureus*. MRSA: methicillin-resistant *Staphylococcus aureus*. ND: not determined. Standard antibiotics were used as positive controls, whereby trimethoprim (1.25 μg) + sulfamethoxazole (23.75 μg) acted as the control for both MRSA isolates, *S. epidermidis*, *S. saprophyticus*, *P. vulgaris*, and *P. mirabilis*; penicillin G (10 μg) for MSSA; erythromycin (15 μg) for *B. cereus* and *B. subtilis*; gentamicin (10 μg) for *A. baumannii* and *E. clocae*; teicoplanin (30 μg) for *E. faecalis*, *E. casseliflavus*, and *E. gallinarum*; and linezolid (30 μg) was used for *E. faecium*. Test performed in triplicate and repeated three times (n = 3).

**Table 4:** NMR data for compound quercetin-3-O-β-D-glucuronide in deuterated methanol (MeOD).

Carbon Number	^1^H	^13^C	HMBC (^13^C)
1			
2		157.6	
3		134.0	
4		177.8	
4a		104.2	
5		161.6	
6	H (d) 6.10	98.5	C-5, C-7, C-8 and C-4a
7		164.6	
8	H (d) 6.29	93.3	C-6, C-4a, C-8a, and C-7
8a		157.0	
1′		121.4	
2′	H; (s) 7.58	115.9	C-2, C-1′, C-6′, C-3′ and C-4′
3′		144.5	
4′		148.5	
5′	H; (d) 6.74	114.6	C-1′, C-6′, C-4′ and C-3′
6′	H; (d) 7.50	121.9	C-2, C-2′, C-4′ and C-5′
1′′			
2′′	H; (d) 3.64	76.2	
3′′	H; (t) 3.47	71.5	
4′′	H; (t) 3.35	76.2	
5′′	H; (t) 3.42	74.0	C-4′′, and C-6′′
6′′	H; (d) 5.23	102.8	C-3 and C-5′′

^1^H NMR (600 MHz, MeOD) δ 7.58 (s, ^1^H), 7.50 (d, J = 8.5 Hz, ^1^H), 6.74 (d, ^1^H), 6.29 (d, J = 2.0 Hz, ^1^H), 6.10 (d, J = 11.6 Hz, ^1^H), 5.23 (d, ^1^H), 3.64 (d, ^1^H), 3.47 (t, ^1^H), 3.42 (t, ^1^H), 3.35 (t, ^1^H). ^13^C NMR (150 MHz, MeOD) δ 71.5, 74.0, 76.2, 93.3, 98.5, 102.8, 104.2, 114.6, 115.9, 121.4, 121.9, 134.0, 144.5, 148.5, 157.0, 157.6, 161.6, 164.6, 177.8.

**Table 5:** NMR data for compound shikimic acid in deuterated dimethyl sulfoxide (DMSO).

Carbon Number	^1^H	^13^C	HMBC (^13^C)
1		129.5	
2	H (s) 6.58	138.4	C-1, C-4, C-6 and COOH
3	H (s) 4.20	65.9	C-1, C-2 and C-5
4	H (m) 3.52–3.54	70.9	C-2, C-3, C-5 and C-6
5	H (m) 3.81–3.84	67.2	C-1, C-3, C-4 and COOH
6a	H (d) 2.40	30.6	C-1, C-2, C-4, C-5 and COOH
6b	H (d) 1.99	30.6	C-1, C-2, C-4, C-5 and COOH
COOH	-	168.8	-

^1^H NMR (600 MHz, DMSO-d6) δ 6.58 (s, ^1^H), 4.20 (s, ^1^H), 3.52–3.54 (m, ^1^H), 3.81–3.84 (d, ^1^H), 2.40 (d, ^1^H), 1.99 (d, ^1^H). ^13^C NMR (150 MHz, DMSO-d6) δ 30.6, 65.9, 67.2, 70.9, 129.5, 138.4, 168.8.

**Table 6:** MIC values of isolated compounds from *S. hillii* methanol extract.

Bacterial Strain	Antibiotic Standard		Compounds (µg/mL)	
	Name	MIC (µg/mL)	Quercetin	Shikimic Acid
Gram-positive bacteria				
*Bacillus cereus* (ATCC 14579)	Erythromycin	5.0	>200.0	>200.0
MRSA (ATCC 33591)	SXT	5.0 *	>200.0	>200.0
*Enterococcus faecalis* (QUT 1105)	Teicoplanin	30.0 *	0.78	200.0
Gram-negative bacteria				
*Proteus vulgaris* (ATCC 6380)	SXT	5.0	200.0	>200.0
*Acinetobacter baumannii* (ATCC 19606)	Gentamicin	10.0 *	>200.0	>200.0
*Enterobacter cloacae* (ATCC 13047)	Gentamicin	20.0 *	200.0	200.0
*Klebsiella pneumoniae* (ATCC 27736)	Gentamicin	2.0 *	>200.0	>200.0
*Pseudomonas aeruginosa* (ATCC 27853)	Gentamicin	10.0 *	200.0	200.0

Quercetin-3-O-β-D-glucuronide inhibited *E. faecalis* at 0.78 µg/mL, *P. vulgaris* at 200 µg/mL, *P. aeruginosa* at 200 µg/mL, and *E. cloacae* at 200 µg/mL. Shikimic acid inhibited the growth of *E. faecalis*, *E. cloacae*, and *P. aeruginosa* at 200 µg/mL. MIC values were determined by the lack of INT reduction to INT formazan measured at 550 nm and expressed as the mean of triplicates. Test performed in three replicates (n = 3). SXT: trimethoprim + sulfamethoxazole. MSSA: methicillin-sensitive *Staphylococcus aureus*. MRSA: methicillin-resistant *Staphylococcus aureus*. *: MIC values obtained from EUCAST (The European Committee on Anti-microbial Susceptibility Testing).

**Table 7:** Physicochemical properties of quercetin-3-O-β-D-glucuronide.

Descriptors	Values	Recommended Range
Molecular weight	478.36	150–500 g/mol
Num. H-bond acceptors	13	10
Num. H-bond donors	8	5
Molar Refractivity	110.77	40 to 130
TPSA	227.58 A2	20–130 Å^2^
Log Po/w	1.13	<5
GI absorption	Low	-
CYP1A2 inhibitor	No	-
CYP2C19 inhibitor	No	-
CYP2C9 inhibitor	No	-
CYP2D6 inhibitor	No	-
CYP3A4 inhibitor	No	-
Lipinski rule violation	2	Maximum 4

## Data Availability

The data presented in this study are available in Supplementary Materials.

## Supplementary Materials

The following are available online at https://www.mdpi.com/article/10.3390/plants11030283/s1, File 1: Spectroscopic data for quercetin-3-O-β-D-glucuronide and shikimic acid.
