# Supplementary material for: Antibacterial Potential of Extracts and Phytoconstituents Isolated from Syncarpia hillii Leaves In Vitro
Source: Plants (Basel). 2022 Jan 21;11(3):283. doi: 10.3390/plants11030283 (PMC8838964; doi:10.3390/plants11030283)
Supplement: Supplementary file 1 [file plants-11-00283-s001.zip › plants-1544807-supplementary.pdf]

Antibacterial potential of extracts and phytoconstituents isolated from *Syncarpia hillii* leaves *in vitro*

Muthukuttige M.N. Perera[1], Satish N. Dighe[2*], Peter L. Katavic[1], and Trudi A. Collet[1*]

[1]Innovative Medicines Group, Queensland University of Technology, Faculty of Health, 60 Musk Avenue, Kelvin Grove, QLD, Australia 4059

*Correspondence: sdighe2008@gmail.com; t.collet@qut.edu.au

1) Figures

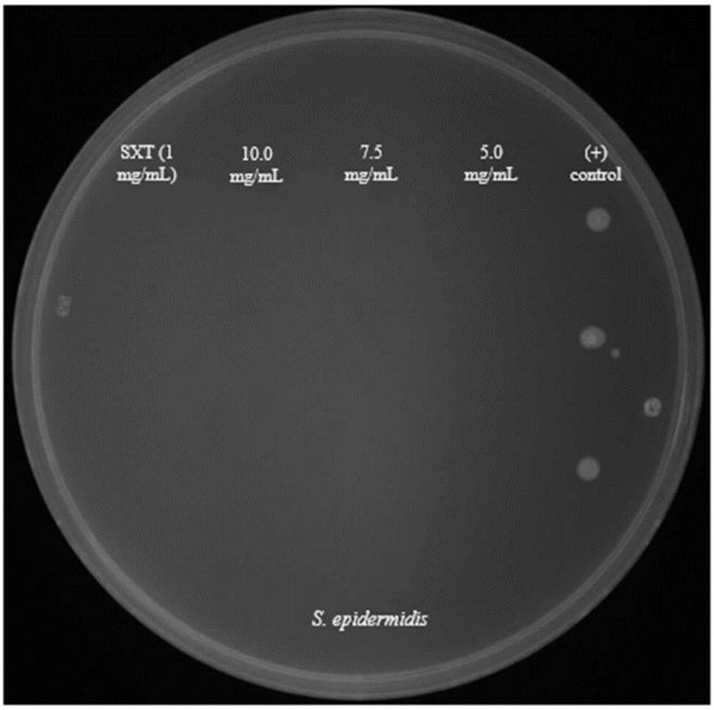

Figure S1. MBC assay for *S. hillii* methanol extract against *Staphylococcus epidermidis*. *S. hillii* methanol extract exhibited bactericidal effects against the bacterium at 5.0 mg/mL. Mueller-Hinton (MH) agar plate layout: 2 μL of 1 mg/mL SXT (Trimethoprim + Sulfamethoxazole), *S. hillii* extract (MBC at 5 mg/mL) and positive control (*S. epidermidis* in MH broth) in triplicate were placed onto the surface of a sterile MH agar plate.

2) Spectroscopic data for Quercetin-3-O-β-D-glucuronide

I) NMR spectra of Quercetin-3-O-β-D-glucuronide

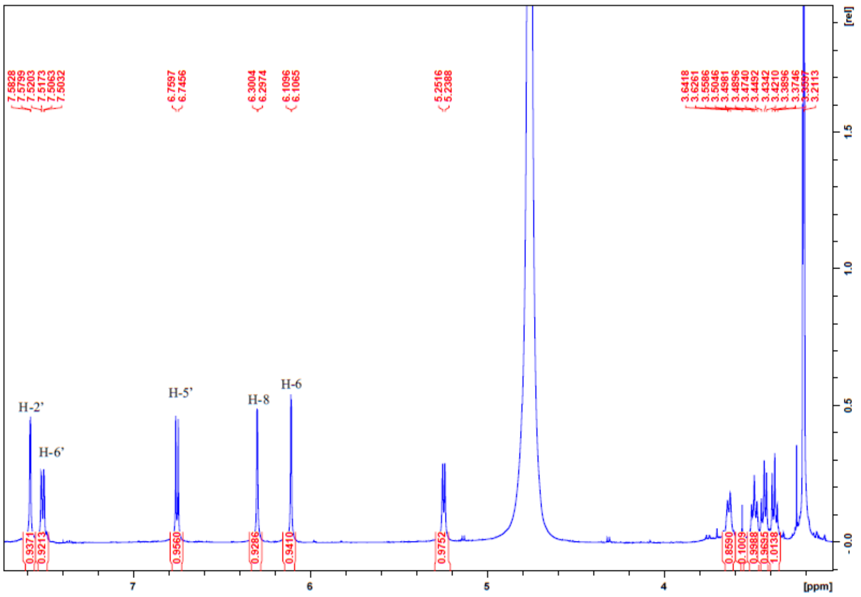

Figure S2. ^{1}H nuclear magnetic resonance (NMR) spectrum (600 Hz, Deuterated methanol: CD3OD-d4) of Quercetin-3-O-β-D-glucuronide.

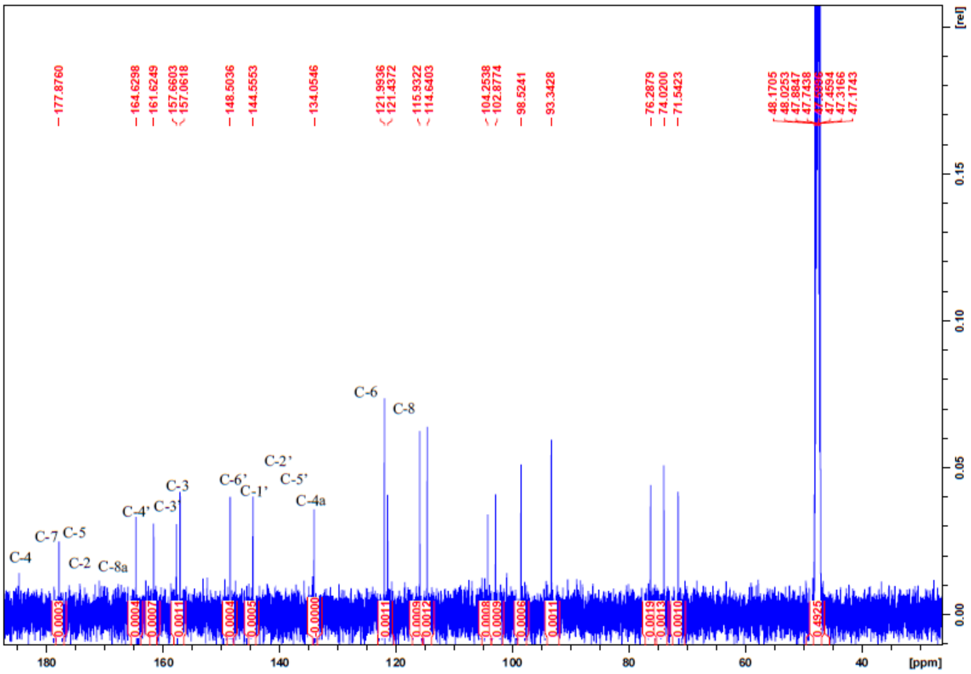

Figure S3. ^{13}C nuclear magnetic resonance (NMR) spectrum (600 Hz, Deuterated methanol: CD3OD-d4) of Quercetin-3-O-β-D-glucuronide.

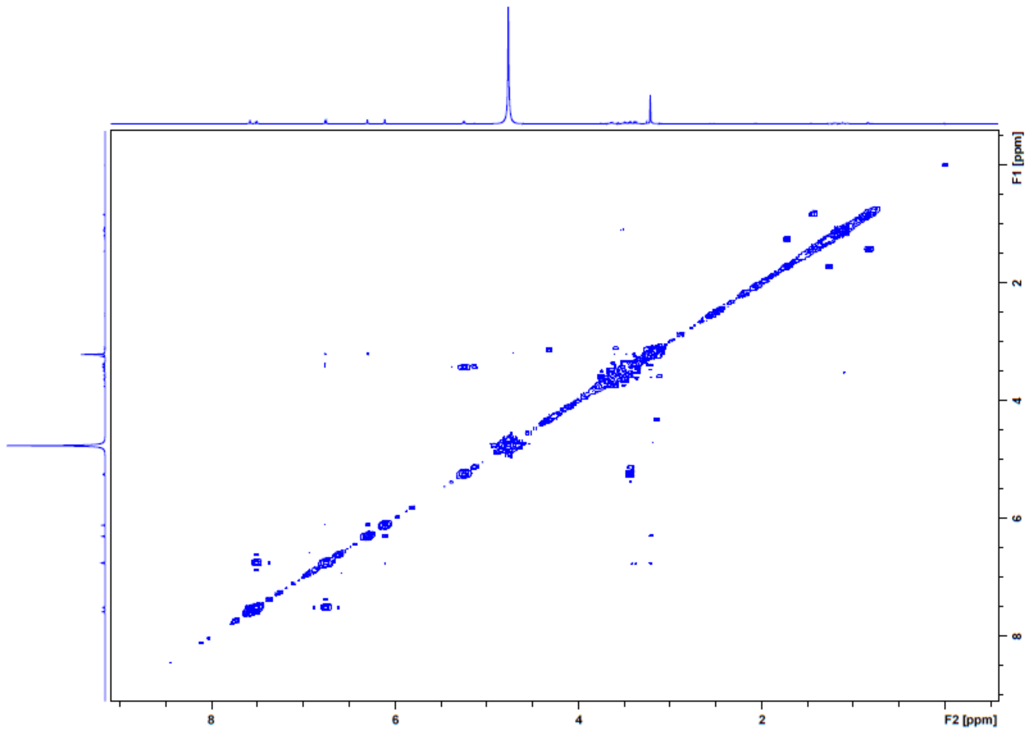

Figure S4. Two-dimensional nuclear magnetic resonance spectroscopy: correlated spectroscopy (COSY) spectrum (600 Hz, Deuterated methanol: CD3OD-d4) of Quercetin-3-O-β-D-glucuronide.

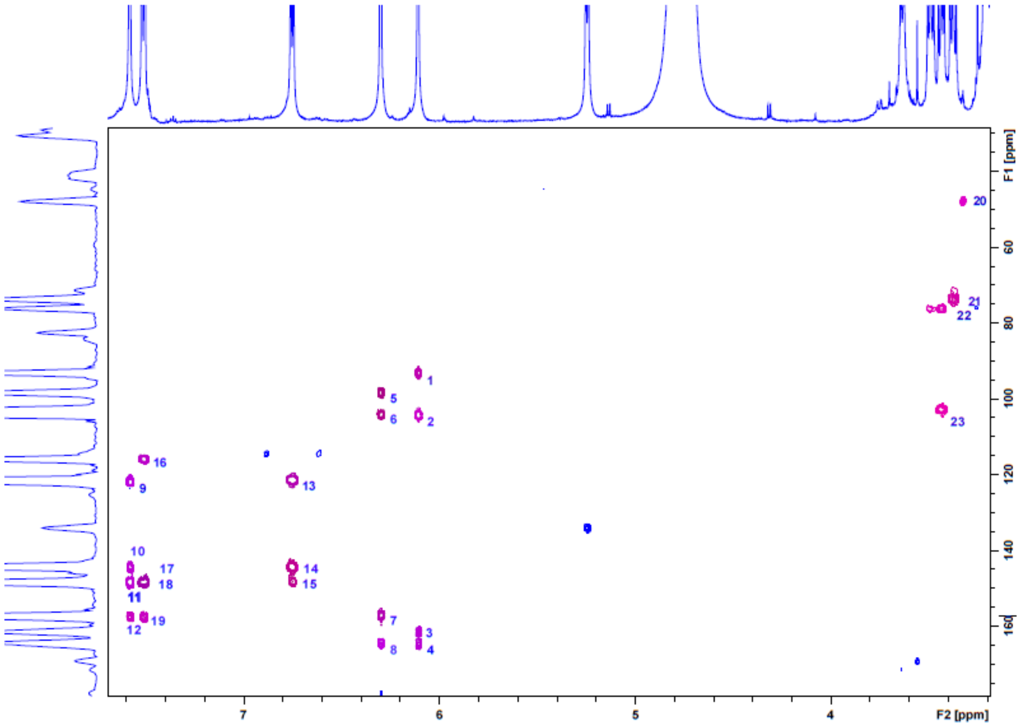

Figure S5. Two-dimensional nuclear magnetic resonance spectroscopy: heteronuclear multiple bond correlation (HMBC) spectrum (600 Hz, Deuterated methanol: CD3OD-d4) of Quercetin-3-O-β-D-glucuronide.

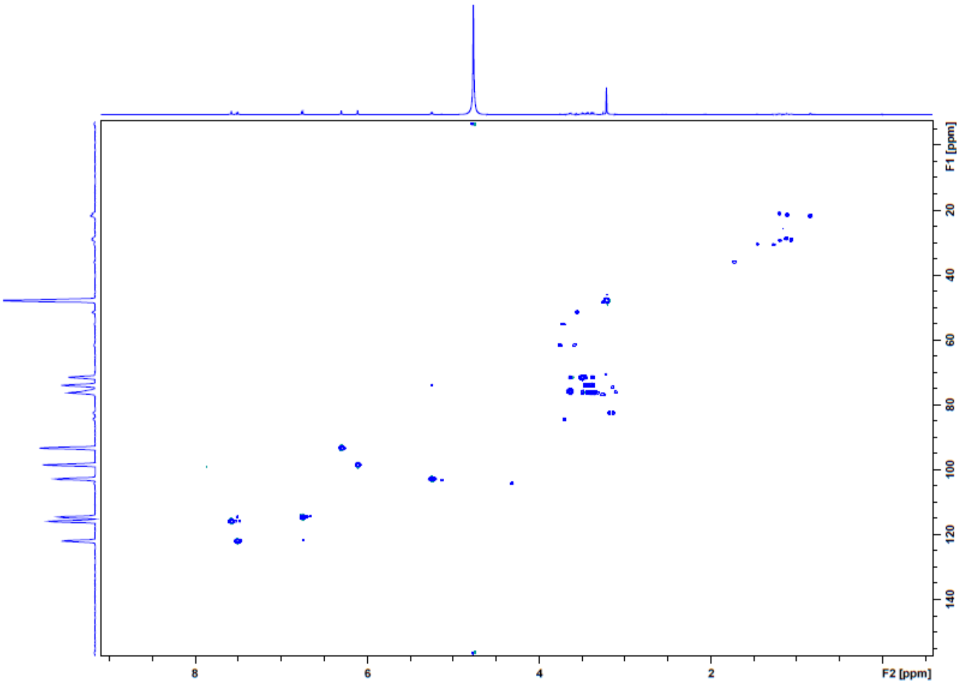

Figure S6. Two-dimensional nuclear magnetic resonance spectroscopy: heteronuclear single quantum correlation (HSQC) spectrum (600 Hz, Deuterated methanol: CD3OD-d4) of Quercetin-3-O-β-D-glucuronide.

II) Mass spectra of Quercetin-3-O-β-D-glucuronide

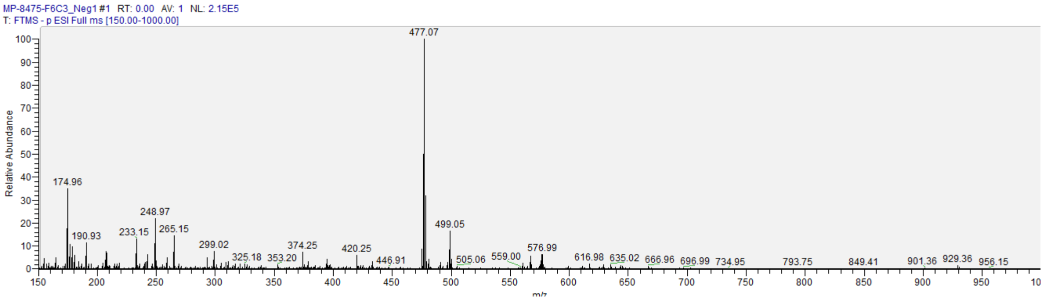

Figure S7. Mass spectrum (MS) in negative ion mode (m/z 150–1000, scan rate of 0.5 Hz, voltage 2.0 kV) of Quercetin-3-O-β-D-glucuronide.

3) Spectroscopic data for Shikimic acid

I) NMR spectra of Shikimic acid

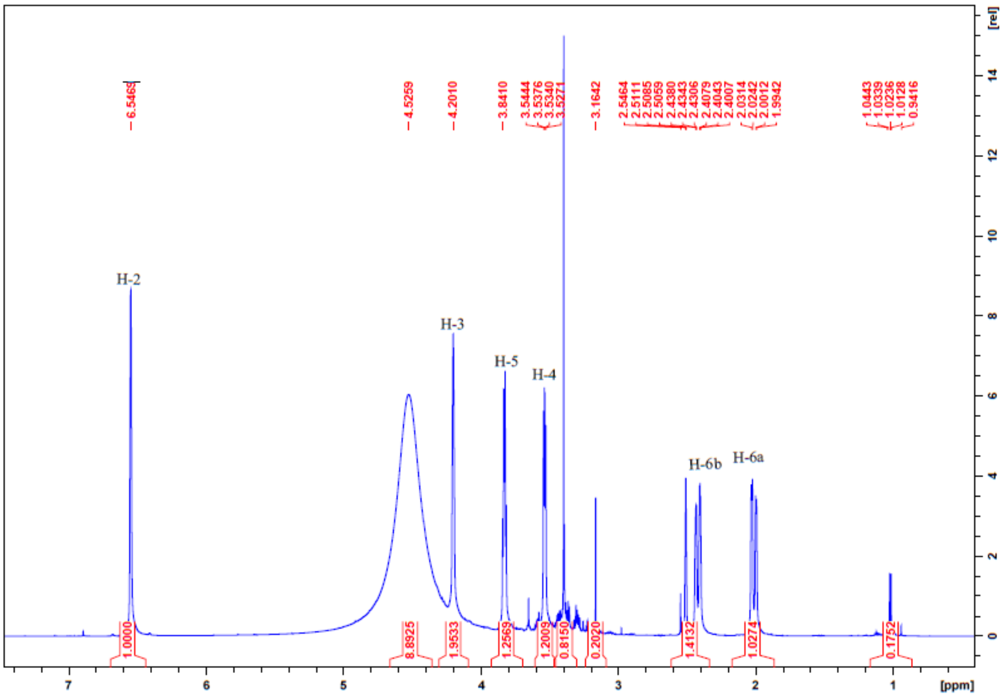

Figure S8. ^{1}H nuclear magnetic resonance (NMR) spectrum (600 Hz, Deuterated dimethyl sulfoxide: DMSO-d6) of Shikimic acid.

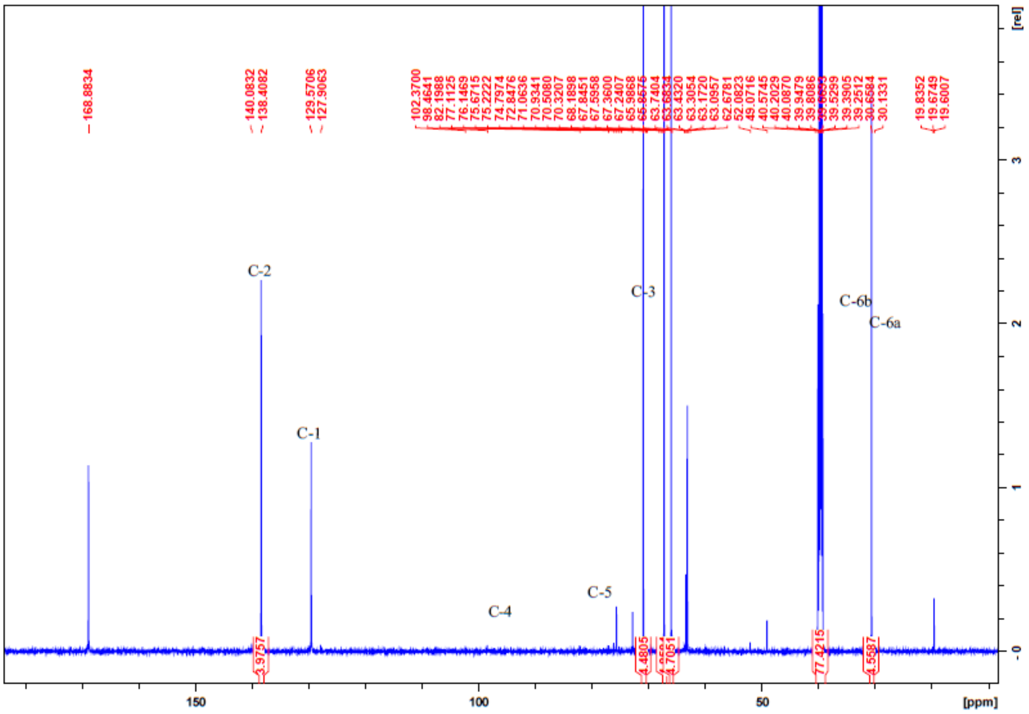

Figure S9. ^{13}C nuclear magnetic resonance (NMR) spectrum (600 Hz, Deuterated dimethyl sulfoxide: DMSO-d6) of Shikimic acid.

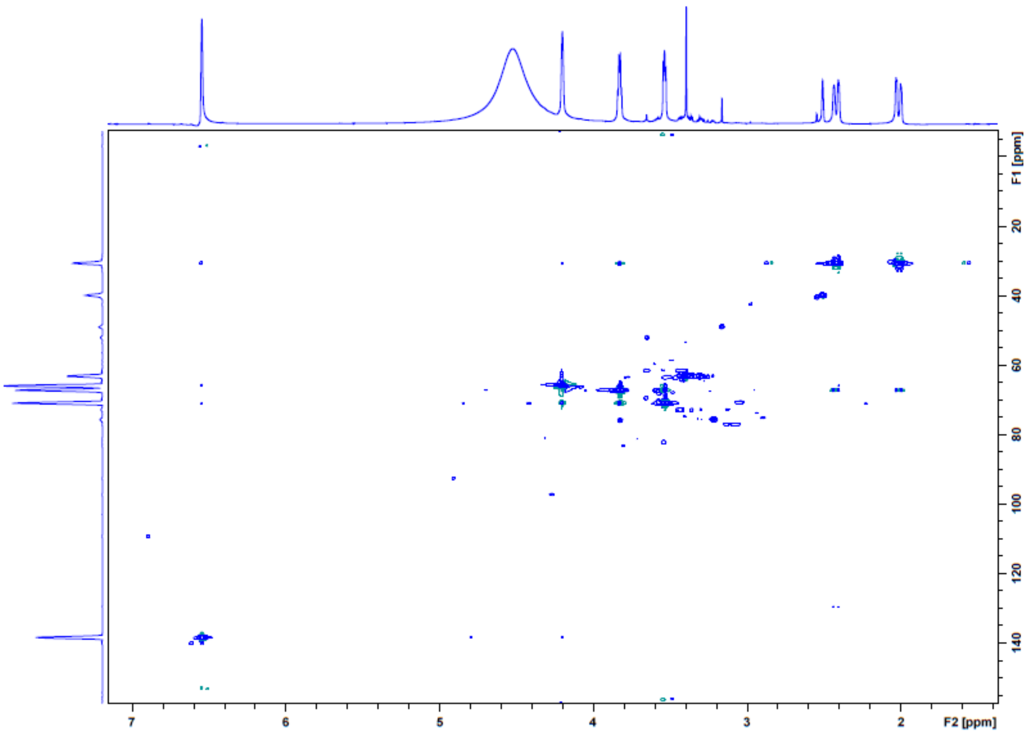

Figure S10. Two-dimensional nuclear magnetic resonance spectroscopy: heteronuclear single quantum correlation (HSQC) spectrum (600 Hz, Deuterated dimethyl sulfoxide: DMSO-d6) of Shikimic acid.

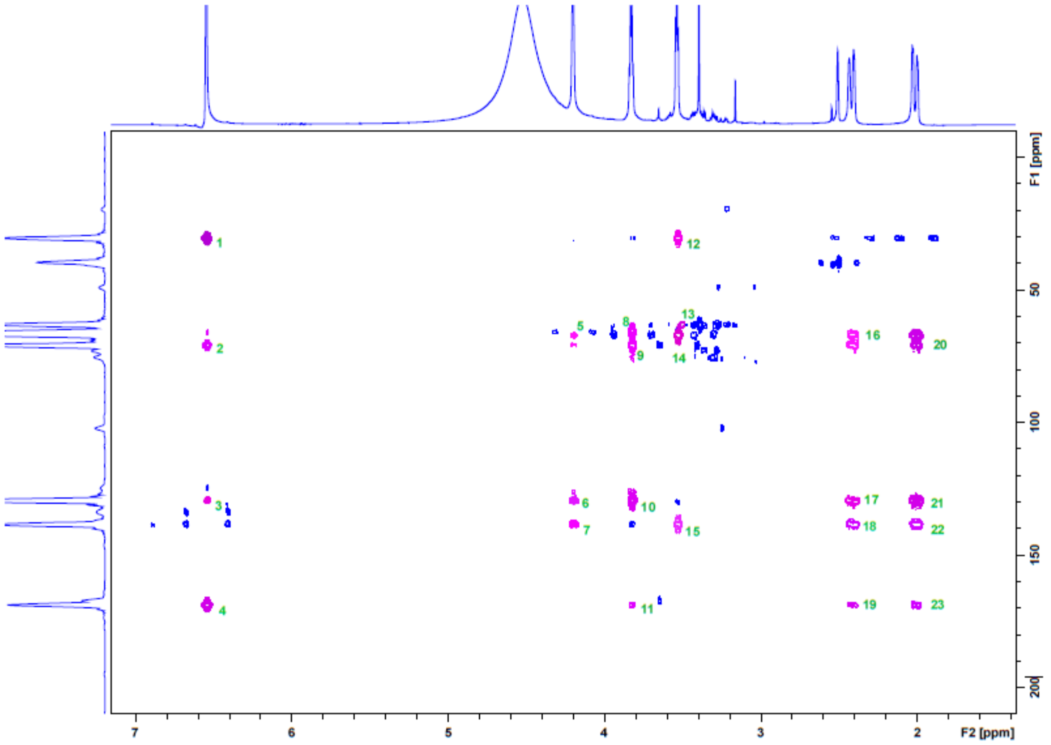

Figure S11. Two-dimensional nuclear magnetic resonance spectroscopy: heteronuclear multiple bond correlation (HMBC) spectrum (600 Hz, Deuterated dimethyl sulfoxide: DMSO-d6) of Shikimic acid.

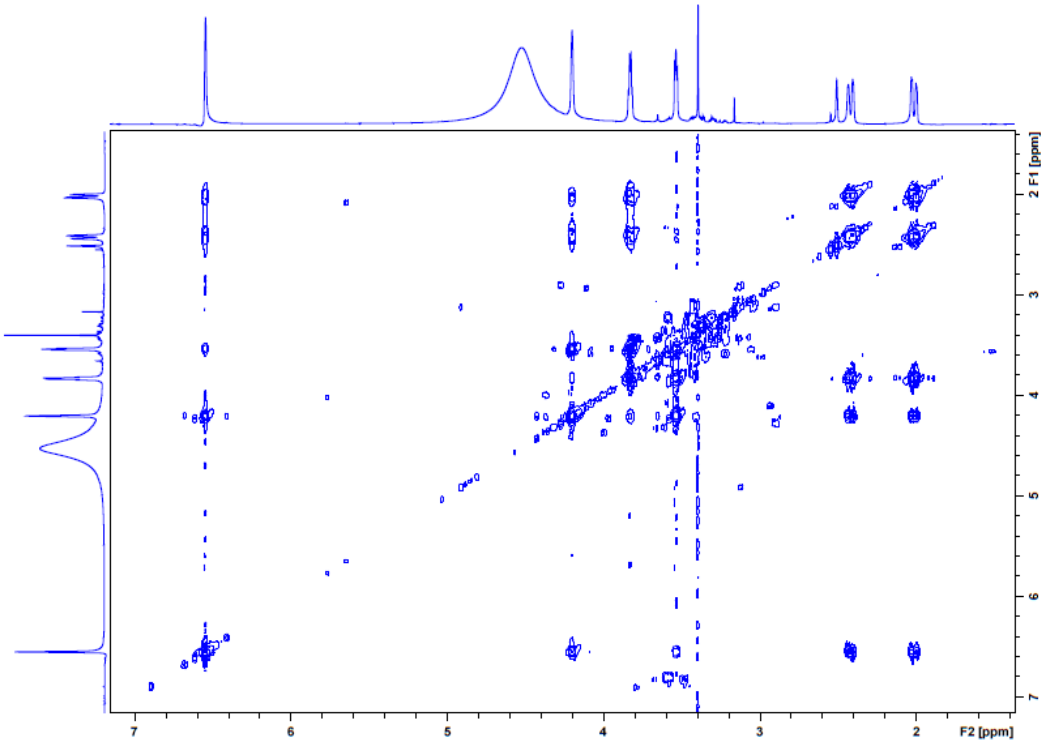

Figure S12. Two-dimensional nuclear magnetic resonance spectroscopy: correlated spectroscopy (COSY) spectrum (600 Hz, Deuterated dimethyl sulfoxide: DMSO-d6) of Shikimic acid.

B) Mass spectra of Shikimic acid

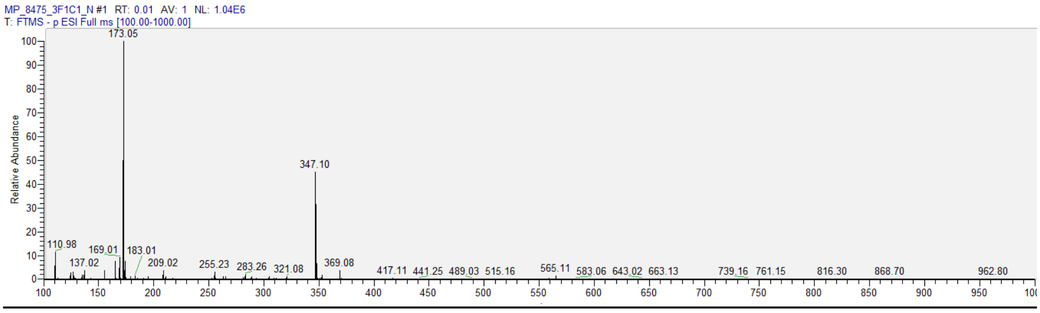

Figure S13. Mass spectrum (MS) in negative ion mode (m/z 150–1000, scan rate of 0.5 Hz, voltage 2.0 kV) of Shikimic acid.
